# Supplementary material for: Genetic Profiling of Cell-Free DNA in Liquid Biopsies: A Complementary Tool for the Diagnosis of B-Cell Lymphomas and the Surveillance of Measurable Residual Disease
Source: Cancers (Basel). 2023 Aug 8;15(16):4022. doi: 10.3390/cancers15164022 (PMC10452485; doi:10.3390/cancers15164022)
Supplement: Supplementary file 1 [file cancers-15-04022-s001.zip › cancers-2520886-supplementary.pdf]

Article

Genetic Profiling of Cell-Free DNA in Liquid Biopsies: A Complementary Tool for the Diagnosis of B-Cell Lymphomas and the Surveillance of Measurable Residual Disease

Gloria Figaredo *, Alejandro Martín-Muñoz, Santiago Barrio, Laura Parrilla, Yolanda Campos-Martín, María Poza, Laura Rufian, Patrocinio Algara, Marina De La Torre, Ana Jiménez Ubieto, Joaquín Martínez-López, Luis-Felipe Casado and Manuela Mollejo

Table S1. Genes included in the panel at diagnosis.

ARID1A	***CXCR4***	***NOTCH1***
ARID1B	*EP300*	*P2RY8*
B2M	*ETS1*	*PAX5*
BCL10	*EZH2*	*PCB1*
BCL2	*FOXO1*	*PIM1*
BCL7A	*GNA13*	*PIM2*
BRAF	*HIST1H1E*	*PRDM1*
BTG1	*HNRNPK*	*RRAGC*
BTK	*ID3*	*S1PR2*
CARD11	*IKZF3*	*SMARCA4*
CCND3	*IRF4*	*SOCS1*
CD58	*IRF8*	*STAT3*
CD79A	*ITPKB*	*STAT6*
CD79B	*KMT2D*	*TCF3*
CDKN2A	*MEF2B*	*TNFAIP3*
CDKN2B	*MFHAS1*	*TNFRSF14*
CIITA	*MUM1*	*TP53*
CREBBP	*MYC*	*XPO1*
CTSS	*MYD88*	
